# Rediscovery and reexamination of a declared 'extinct' plant in South China: The species delimitation and conservation implications of *Callicarpa oligantha* (Lamiaceae)

**DOI:** 10.3897/phytokeys.278.203604

**Published:** 2026-08-17

**Authors:** Huimin Cai, Qiyi Yang, Yicheng Xu, Tieyao Tu, Zhonghui Ma

**Affiliations:** 1 National Demonstration Center for Experimental Plant Science Education, College of Agriculture, Guangxi University, Nanning 530004, Guangxi, China State Key Laboratory of Plant Diversity and Specialty Crops, Guangdong Provincial Key Laboratory of Applied Botany, South China Botanical Garden, Chinese Academy of Sciences Guangzhou China; 2 State Key Laboratory of Plant Diversity and Specialty Crops, Guangdong Provincial Key Laboratory of Applied Botany, South China Botanical Garden, Chinese Academy of Sciences, Guangzhou 510650, China National Demonstration Center for Experimental Plant Science Education, College of Agriculture, Guangxi University Nanning China; 3 Luofu Mountain State-owned Forest Farm, Huizhou 516100, China Luofu Mountain State-owned Forest Farm Huizhou China

**Keywords:** *
Callicarpa
*, conservation, extinct, phylogenetics species delimitation, synonym, taxonomy

## Abstract

Accurate species delimitation serves as a cornerstone for effective biodiversity conservation strategies, particularly for rare taxa where misclassification of taxa may lead to misallocation of limited resources. *Callicarpa oligantha*, one of only two plant species declared extinct in Guangdong Province, South China, was historically documented solely from its type specimen--a single individual collected in 1917 at Luofushan (Loh Fau Mountain). However, the morphological similarities with *C. brevipes* have long challenged the taxonomic status of *C. oligantha*. Following the rediscovery of the species at its type locality in 2024, we conducted an integrative taxonomic reassessment of *C. oligantha* and *C. brevipes* based on the newly collected materials using combined analyses of molecular phylogenetics and morphological comparisons. Both molecular and morphological evidence congruently support the conspecificity of *C. oligantha* and *C. brevipes*. We therefore propose synonymizing *C. oligantha* under *C. brevipes* and recommend its exclusion from extinction lists. This study underscores the importance of robust taxonomic frameworks in preventing conservation misprioritization, and highlights the value of integrative, multidisciplinary approaches for establishing accurate biodiversity baselines prior to biological conservation action.

## Introduction

The precise delineation of species boundaries and accurate identification form the foundation of effective biodiversity conservation, particularly for rare and taxonomically ambiguous taxa characterized by limited research (Li et al. 2023; Lin et al. 2024; Hong 2025). Therefore, without clear knowledge of where species limits should be set, we are unable to properly assess the threatened status of species and develop rational conservation plans and management (Mace 2004). More critically, erroneous classification and misidentification may exclude genuinely endangered species from protection, while management actions based on incorrect species identification would inevitably waste limited conservation resources and funding (Mace 2004; Feng et al. 2021; Ji et al. 2021). For instance, *Fagus chienii* Cheng was designated as a key protected species in 2016 as no additional specimens have been collected since its initial discovery based on a single collection in 1935. However, recent molecular and morphological evidence has clearly demonstrated that this purportedly endangered plant (*F. chienii*) is conspecific with *F. hayatae* Palib. (Li et al. 2023). Consequently, Li et al. (2023) recommend that its protection strategy should be canceled in conservation management. Another notable case is Torreya grandis var. jiulongshanensis Zhi Y.Li, Z.C.Tang & N.Kang, which was listed as a Category II protected plant recorded in the List of National Key Protected Wild Plants (https://www.forestry.gov.cn/c/www/lczc/10746.jhtml). Nevertheless, Kou et al. (2017) conducted a phylogenetic study and clearly demonstrated that this endangered plant was a natural hybrid between *T. jackii* Chun and *T. grandis* Fortune ex Lindl., challenging the rationale for its protected status. These cases collectively affirm that effective conservation strategies for endangered species must be grounded in rigorous research and well-defined species delimitations to guide appropriate management and restoration efforts.

*Callicarpa* L., with the nickname 'beauty berry', was initially recognized by Linnaeus (1753) for its distinctive purple fruits. It encompasses approximately 162 species (Plants of the World Online, https://powo.science.kew.org/) found in temperate, subtropical and tropical regions of Asia, Australia and America, exhibiting a typical amphi-Pacific distribution pattern (Leeratiwong et al. 2007; Bramley 2013; Cai et al. 2023). There are *ca*. 48 species recorded in China, predominantly distributed in the regions south of the Yangtze River. Within this genus, *C. oligantha* Merr. is a species with an exceptionally narrow distribution, recorded at Luofushan (Loh Fau Mountain) in 1918, Guangdong Province, China (Merrill 1918; Chen and Michael 1994). Merrill (1918) provided an original description of *C. oligantha* based on a gathering from a single individual (No. 11060, NY and US) and noted that the alliance of this species was manifestly with *C. dichotoma* (Lour.) K.Koch. However, *C. oligantha* distinctly differs from *C. dichotoma* in relatively much longer and narrower leaves, subsessile, abaxially pubescent, shorter filament length and very few-flowered cymes. Since *C. oligantha*'s original description based on a single collection in 1917, no additional individuals of *C. oligantha* have been documented for nearly 110 years. Therefore, it was even listed as "possibly extinct" in the Provincial Red List of Higher Plants in Guangdong Province (Wang 2022). However, as an endemic species in China, *C. oligantha* has not yet been assessed in the IUCN Red List of Threatened Species (IUCN 2025) and it was categorized as ‘data deficient’ in the Red List of China's Biodiversity: Higher Plants (2020) (Ministry of Ecology and Environment of the People's Republic of China, and Chinese Academy of Sciences 2023). In Chang's (1951) review of the Chinese species of *Callicarpa*, *C. oligantha* was considered closely allied to *C. brevipes* (Benth.) Hance, with suggestions that it might represent a morphological variant of the latter (Chang 1951). In light of this, we reexamined the type specimens of *C. oligantha* and also considered it was morphologically extremely similar to *C. brevipes*. During a field survey at the Luofu Mountain, we rediscovered a population of *Callicarpa* that closely matches the original morphological description of *C. oligantha*, except for the presence of pubescent fruits and occasional pubescence on the abaxial leaf surfaces in some individuals. These variations were not mentioned in the original species description of *C. oligantha*, likely because Merrill had accessed only a single specimen from one individual at that time. This limited material may have contributed to the current taxonomic uncertainty of the species.

Currently, resolving the enigmatic status of *Callicarpa oligantha* is imperative for conservation. To clarify *C. oligantha*’s taxonomic status and provide a more objective conservation treatment, we used the recently collected samples to reconstruct its phylogenetic position based on nuclear ribosomal internal and external transcribed spacer regions (nrITS and nrETS) and eight chloroplast DNA (cpDNA) markers. We also determined whether *C. oligantha* is morphologically distinct from its close relative (*C. brevipes*) through statistical analyses of six morphological characters. Accurately delimiting species boundaries is critically important in conservation biology, as taxonomic inaccuracies may profoundly compromise downstream inferences. Effective protection of endangered species should be based on thoroughly researched and well-supported species delimitations.

## Materials and methods

### Phylogenetic analyses

Twenty species spanning a broad phylogenetic frame of *Callicarpa* were examined in this study, with *Dicrastylis parvifolia* F.Muell. and *Dasymalla teckiana* (F.Muell.) B.J.Conn & Henwood as outgroups and voucher information of the samples are shown in Table 1. Five individuals of *C. oligantha* collected from type locality were included. To enlarge the intraspecific representation of *C. brevipes*, which was assumed to be conspecific with *C. oligantha*, three *C. brevipes* individuals were included. Extracting total genomic DNA of the samples with silica dried leaf tissue followed the 2× CTAB method of Doyle and Doyle (1987). According to Ma (2013) and Cai et al. (2023), two nuclear ribosomal regions (nrITS and nrETS) and eight cpDNA regions (*matK*, *psbJ-petA*, *rpl32-trnL*, *trnD-trnT*, *trnH-psbA*, *trnQ-5'rps16*, *trnS (GCU)-trnG* and *3'trnV-ndhC* intergenic spacer) are considered effective in phylogenetic reconstructions of *Callicarpa*. They were amplified and primer pairs used in polymerase chain reaction (PCR) amplification which are listed in Suppl. material 1: table SS1 (White et al. 1990; Sun et al. 1994; Demesure et al. 1995; Sang et al. 1997; Baldwin and Markos 1998; Beardsley and Olmstead 2002; Tate and Simpson 2003; Shaw et al. 2007; Andersson 2006; Shaw et al. 2007; Dong et al. 2012). The PCR products were sequenced using Sanger sequencing on the BGI’s platform with both directions to ensure accuracy. The new sequence data of each locus were deposited in GenBank (http://www.ncbi.nlm.nih.gov) and the accession numbers were shown in Table 2 (* indicates sequences newly generated in this study).

**Table 1.** DNA samples information for phylogenetic analyses of *Callicarpa oligantha* and *C. brevipes* in this study.

**Species**	**Voucher**	**Collector**	**Location**	**DNA No**.
*C. americana*	K-LCD_081-84-00-507 (K)	#	#	14293
*C. longifolia*	H. Toyama et al. 2329 (Kyushu University)	Toyama et al.	Kampot, Cambodia	2329
*C. pentandra*	Bramley et al. SAN1472 (K)	Bramley et al.	Bombalai Hill, Malaysia	25527
*C. pentandra*	Leeratiwong 06-333 (KKU)	Leeratiwong	Narathiwat, Thailand	38170
*C. furfuracea*	Leeratiwong 06-325 (KKU)	Leeratiwong	Songkhla, Thailand	38163
*C. angustifolia*	H. Toyama et al. 774 (Kyushu University)	Toyama et al.	Kho Khong, Cambodia	774
*C. candicans*	LB 0162 (IBSC)	Li B	Hainan, China	MZH20
*C. cathayana*	X.X.Huang 8918 (IBSC)	Huang XX	Jiangxi, China	MZH54
*C. dichotoma*	ZHM 080 (IBSC)	Ma ZH	Guangdong, China	MZH2
*C. erythrosticta*	X.X.Huang 8700 (IBSC)	Huang XX	Jiangxi, China	MZH68
*C. pedunculata*	ZHMa 083 (IBSC)	Ma ZH	Guangdong, China	MZH1
*C. japonica*	ZHMa 0160 (IBSC)	Ma ZH	Korea	MZH35
*C. kochiana*	LB (IBSC)	Li B	Guangdong, China	MZH49
*C. loboapiculata*	ZHMa 013 (IBSC)	Ma ZH	Guangxi, China	MZH14
*C. rubella*	ZHMa 085 (IBSC)	Ma ZH	Guangdong, China	MZH9
*C. luteopunctata*	G. Yao 402 (IBSC)	Yao G	Sichuan, China	MZH53
C. integerrima var. chinensis	X.X. Huang 1032 (IBSC)	Huang XX	Hunan, China	MZH60
*C. rubella*	ZHMa 089 (IBSC)	Ma ZH	Guangdong, China	MZH6
*C. arborea*	ZHMa 096 (IBSC)	Ma ZH	Yunnan, China	MZH75
*C. brevipes*	ZHMa 0130 (IBSC)	Ma ZH	Guangdong, China	MZH44
*C. brevipes*	5476 (IBSC)	Ma ZH	Hainan, China	MZH46
*C. brevipes*	S. Tagane et al. V3825 (Kyushu University)	Tagane et al.	Ha Tinh, Vietnam	V3825
*C. oligantha*	WeiCX11 (GAUA)	Wei et al.	Guangdong, China	WeiCX11
*C. oligantha*	WeiCX12 (GAUA)	Wei et al.	Guangdong, China	WeiCX12
*C. oligantha*	WeiCX13 (GAUA)	Wei et al.	Guangdong, China	WeiCX13
*C. oligantha*	WeiCX14 (GAUA)	Wei et al.	Guangdong, China	WeiCX14
*C. oligantha*	ZYC312 (GAUA)	Zhang et al.	Guangdong, China	ZYC312
*C. kwangtungensis*	MZH73 (GAUA)	Ma ZH	Guangdong, China	MZH73
*Dasymalla teckiana*	#	#	Australian National Botanic Gardens, Australia	#
*Dicrastylis parvifolia*	#	#	Australian National Botanic Gardens, Australia	#

**Table 2.** List of taxa for phylogenetic analyses with information related to the accession numbers of each locus.

**Taxa**	**ITS**	**ETS**	***matk***	***trnQ*-*rps16***	***psbJ*-*petA***	***rpL32*-*trnL***	***trnD*-*T***	***trnG*-*trnS***	***trnH*-*psbA***	***trnV*-*ndhC***
*C. americana* 14293	ON820115	ON931484	OP032108	OP734977	#	#	OP734891	OP735028	OP735071	OP744560
*C. furfuracea* 38163	OM638741	OM307526	OM630217	OM403917	OM460819	OM501609	OM403750	OM403833	OM473326	OM307468
*C. angustifolia* 774	OM333873	OM307572	OM630205	OM403976	OM489741	OM530204	OM403809	OM403893	OM460777	OM307495
*C. arborea* MZH75	ON820120	ON931489	OP032113	OP734976	OP081535	OP734845	OP734896	OP735027	OP735070	OP744559
*C. candicans* MZH20	OM333844	OM307537	OM630169	OM403924	OM460814	OM501614	OM403757	OM403840	OM473348	OM307476
*C. cathayana* MZH54	ON820161	ON931538	OP032149	OP734957	OP081576	OP734885	OP734939	OP735009	#	OP744545
*C. luteopunctata* MZH53	OM333858	OM307551	OM630179	OM403937	OM460804	OM501627	OM403769	OM403853	OM489766	OM403727
*C. pentandra* 25527	ON820150	ON931526	OP032141	OP734946	OP032158	OP081588	OP734930	OP032169	OP032164	OP744535
*C. pentandra* 38170	OM333837	OM307529	OM630165	OM403918	OM460818	OM501610	OM403751	OM403829	OM473329	OM307469
*C. dichotoma* MZH2	OM333846	OM307539	OM630171	OM403926	OM460812	OM501616	OM403759	OM403842	OM473338	OM307478
C. integerrima var. chinensis MZH60	ON820131	ON931501	OP032123	OP734972	OP081545	OP734853	OP734909	OP735022	OP735066	OP744557
*C. kochiana* MZH49	ON820134	ON931506	OP032126	OP734960	OP081549	OP734857	OP734912	OP735012	OP735055	OP744548
*C. loboapiculata* MZH14	OM333854	OM307547	OM630176	OM403933	OM460807	OM501623	OM403765	OM403849	OM473344	OM403723
*C. longifolia* 2329	OM333883	OM307592	OM630203	OM403972	OM489745	OM530172	OM403805	OM403889	OM460781	OM307512
*C. erythrosticta* MZH68	OM333847	OM307540	OM630172	OM403927	PV718150	OM501617	OM403760	OM403843	OM489770	OM403729
*C. pedunculata* MZH1	OM333848	OM307541	#	OM403928	OM460811	OM501618	OM403761	OM403844	OM473337	OM307479
*C. japonica* MZH35	OM333851	OM307544	#	OM403931	OM460808	OM501621	#	OM403847	OM489762	OM307480
*C. kwangtungensis* MZH73	OM333853	OM307546	OM630175	OM403932	OM530164	OM501622	OM403764	OM403848	OM489773	OM307482
*C. rubella* MZH9	OM333856	OM307549	OM630178	OM403935	OM460805	OM501625	OM403767	OM403851	OM473340	OM403721
*C. rubella* MZH6	OM333864	OM307557	ON964474	OM403988	OP032159	OM530212	OM403821	OM403906	OM473339	PV710156
*C. brevipes* MZH46	OM333843	OM307536	OM630168	OM403923	OM460815	OM501613	OM403756	OP032173	OM489765	OM307475
*C. brevipes* MZH44	ON820123	ON931491	OP032115	#	#	OP081586	OP734900	#	OP032167	#
*C. brevipes* V3825	OM333877	OM307583	OM630196	OM403961	OM460794	OM530197	OM403796	OM403878	OM489799	OM307504
*C. oligantha* WeiCX12*	PV658358	PV718137	PV710150	PV718143	PV718146	PV710153	PV710168	PV710173	PV710163	PV710158
*C. oligantha* WeiCX14*	PV658356	PV718139	PV718141	PV718144	PV718148	PV710154	PV710169	PV710175	PV710165	PV710160
*C. oligantha* WeiCX13*	PV658355	PV718138	PV710151	#	PV718147	PV718152	PV710171	PV710174	PV710164	PV710159
*C. oligantha* ZYC312*	PV658357	PV718140	PV710152	#	PV718149	PV718153	PV710170	PV710176	PV710166	PV710161
*C. oligantha* WeiCX11*	PV658354	PV718136	#	PV718142	PV718145	PV718151	PV710167	PV710172	PV710162	PV710157
*Dasymalla teckiana*	GQ381188	#	NC_058334	NC_058334	NC_058334	NC_058334	NC_058334	NC_058334	NC_058334	NC_058334
*Dicrastylis parvifolia*	GQ381162	#	NC_058335	NC_058335	NC_058335	NC_058335	NC_058335	NC_058335	NC_058335	NC_058335

* indicates sequences newly generated in this study.

To reduce potential bias in phylogenetic reconstruction, both concatenation-based and coalescent-based methods were applied. Raw chromatograms were manually checked and aligned using Sequencher v.5.4.5 (Gene Codes Corporation, Ann Arbor, MI, USA). Phylogenetic analyses were performed based on the concatenated dataset using maximum likelihood (ML) and Bayesian inference (BI) methods using Phylosuite (Zhang et al. 2020). ML analysis was carried out by running 5, 000 bootstrap replicates with ultrafast parameter in IQ-TREE (Nguyen et al. 2015). BI analysis was conducted in MrBayes (Ronquist et al. 2012) and posterior probabilities were approximated by sampling trees using a variant of the Markov Chain Monte Carlo (MCMC) method. A total of 5,000,000 trees were sampled every 1000 generation. The first 25% of the generations were discarded as "burn-in". Average standard deviation of split frequencies was less than 0.01. The best-fitting nucleotide substitution models were listed in Suppl. material 1: table SS2. All the phylogenetic trees were visualized in iTOL (https://itol.embl.de/) (Letunic and Bork 2024) and edited in Adobe Illustrator 2021. To account for incomplete lineage sorting, which can have a major influence on phylogenetic reconstruction, we used a multispecies coalescent approach in StarBEAST2 v.1.0.0 (Ogilvie et al. 2017) to infer the species tree using two nrDNA loci and eight cpDNA loci respectively. The discordance between the nuclear and plastid phylogenies was visualized using the “cophylo” function of the R package PHYTOOLS (Revell 2011) to minimize crossing lines.

### Morphological studies

**Figure 1.**
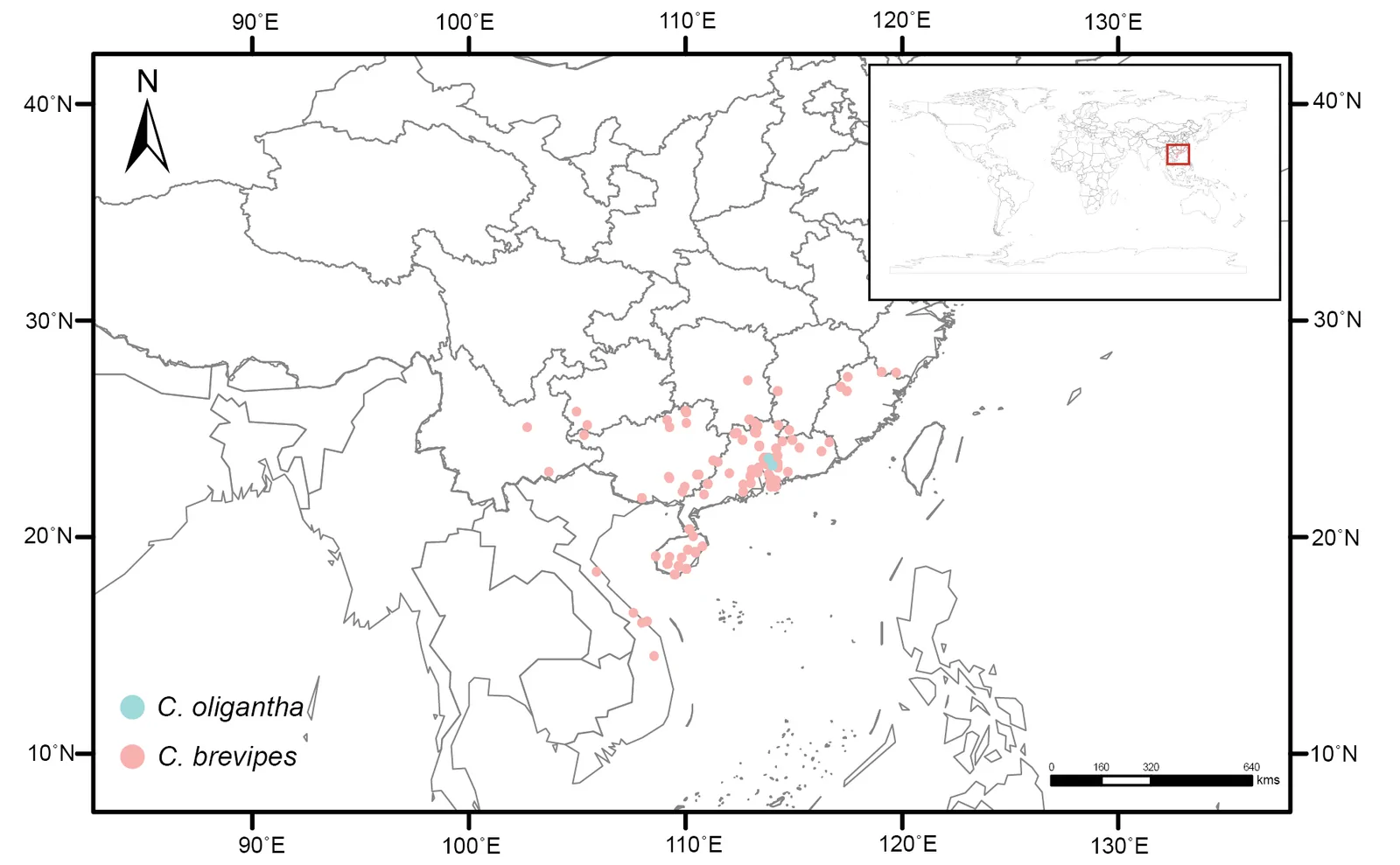
The distribution map of *Callicarpa oligantha* and *C. brevipes*.

For *Callicarpa oligantha*, we examined eight specimens, seven of which were collected in this study from Luofu Mountain (the type locality); the remaining sample was the isotype preserved in the United States National Herbarium (US). Additionally, we measured a total of thirteen specimens of *C. brevipes*, including seven individuals collected in this study (Voucher ID: 6 (Ma ZH), 078 (Ma ZH), 082 (Ma ZH), 0150 (Ma ZH), LB0169 (Li B), 0130 (Ma ZH), 0133 (Ma ZH)), three samples from the Royal Botanic Gardens, Kew (K) (Voucher ID: 12 (Champion, K000674726), 442 (Bentham, K000674725), *s.n*. (Champion, K000674724) and three specimens from South China Botanical Garden Herbarium (IBSC) (Voucher ID: zlx2465 (Zhou et al., IBSC1011661), DSW3838 (Deng and Zeng, IBSC1028900), DSW3931 (Deng SW, IBSC1027227)) (Suppl. material 1: tables S3, S4). The distribution of *C. oligantha* and *C. brevipes* in the world was presented in Fig. 1.

To improve resolution of species clustering, we conducted comparative morphological analyses incorporating the closely related species *Callicarpa kwangtungensis* Chun as an additional reference taxon. Six morphological characters (leaf length, leaf width and leaf petiole length, inflorescence width, inflorescence branching and filament length) assessed as the key characters in distinguishing *Callicarpa* species (Ma 2013), were measured using ImageJ software (https://imagej.net/ij/) (Schneider et al. 2012). Detailed measurements and voucher information are available in Suppl. material 1: tables S3, S4. Principal Component Analysis (PCA) was performed in Origin 2024 (OriginLab Corporation, Northampton, MA, USA) to investigate the relationships between *C. oligantha* and *C. brevipes*. To further test the reliability of the clustering analysis, a Principal Coordinates Analysis (PCoA) using Bray–Curtis dissimilarity index was performed and graphically visualized online in BioLadder (bioladder.cn) (Zhang et al. 2024). The significance level of pairwise comparisons between groups was computed based on Adonis test by *pairwiseAdonis* package in R v.4.4.1 software (Martinez Arbizu 2020). In addition, comparisons of three morphological characters between the two species were conducted and their significances were evaluated by *t*-test using the *ggsignif* package in R v.4.4.1 software (Ahlmann-Eltze and Patil 2021).

## Results

### Molecular phylogenetic relationships of *Callicarpa oligantha* and *C. brevipes*

**Figure 2.**
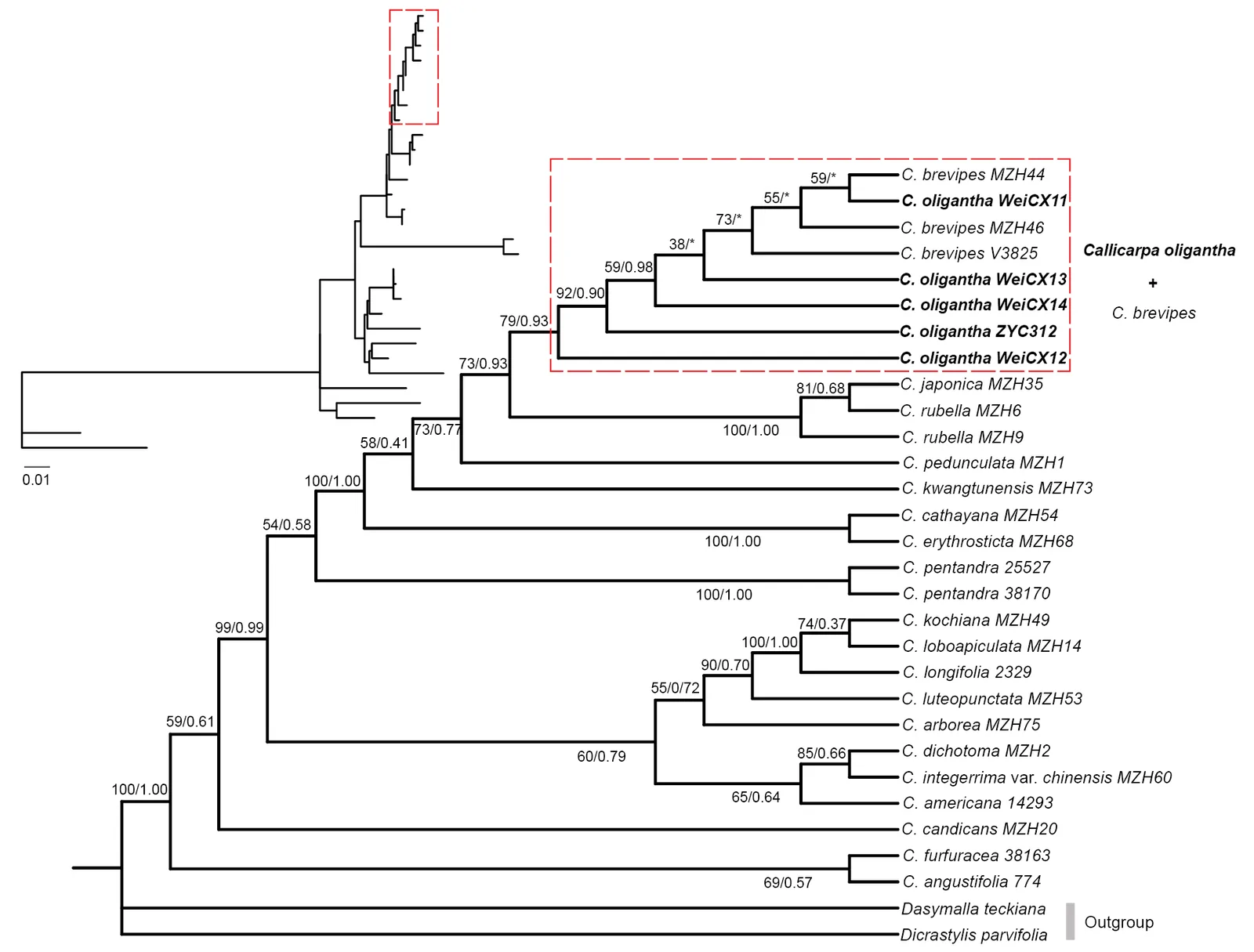
The maximum likelihood (ML) tree based on two nrDNA for the phylogenetic analysis of *Callicarpa oligantha* and *C. brevipes*. Numbers above branches are statistical support values for ML and BI. Bold font represents *C. oligantha*.

**Figure 3.**
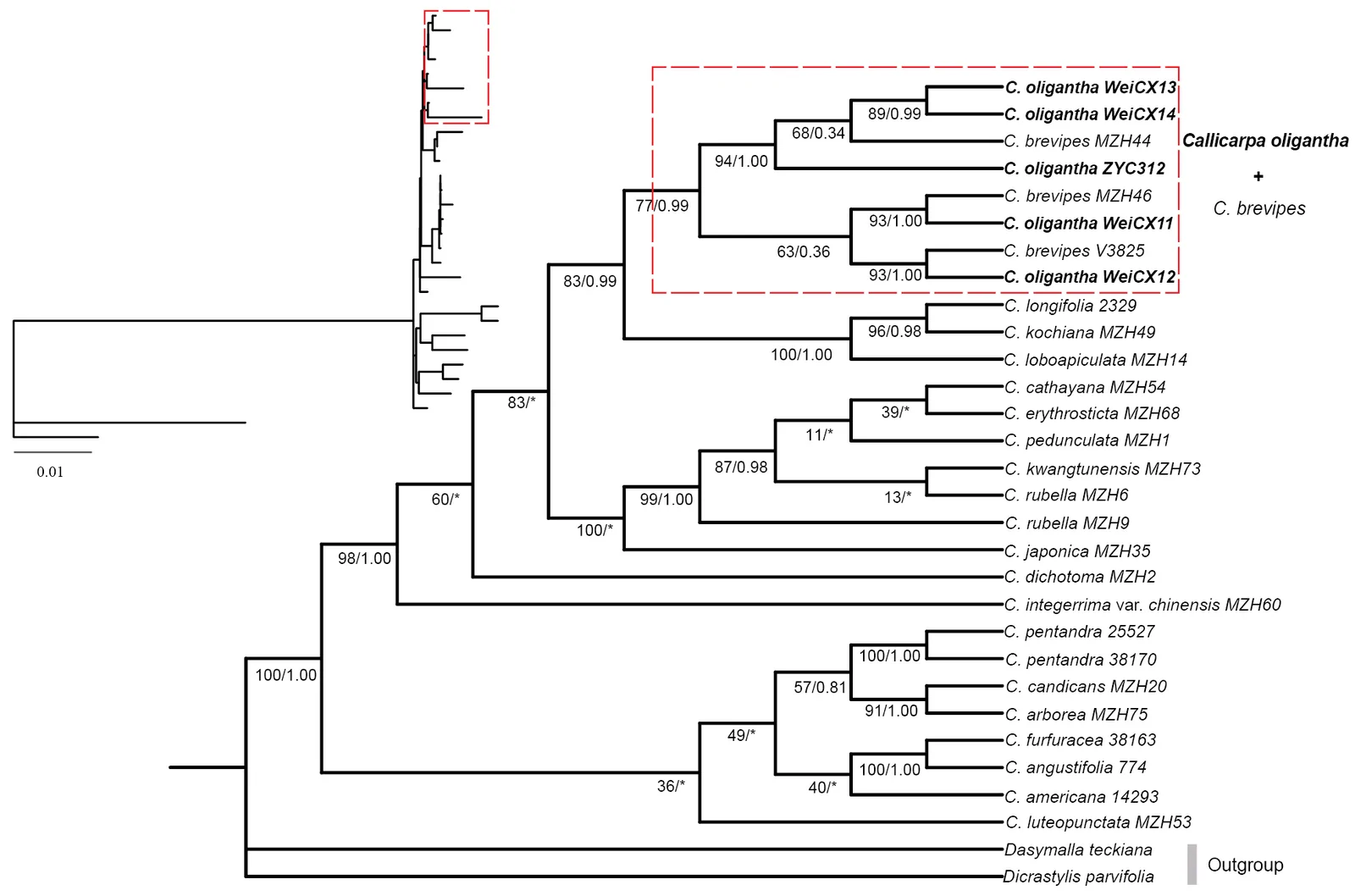
The maximum likelihood (ML) tree based on eight cpDNA for the phylogenetic analysis of *Callicarpa oligantha* and *C. brevipes*. Numbers above branches are statistical support values for ML and BI. Bold font represents *C. oligantha*.

**Figure 4.**
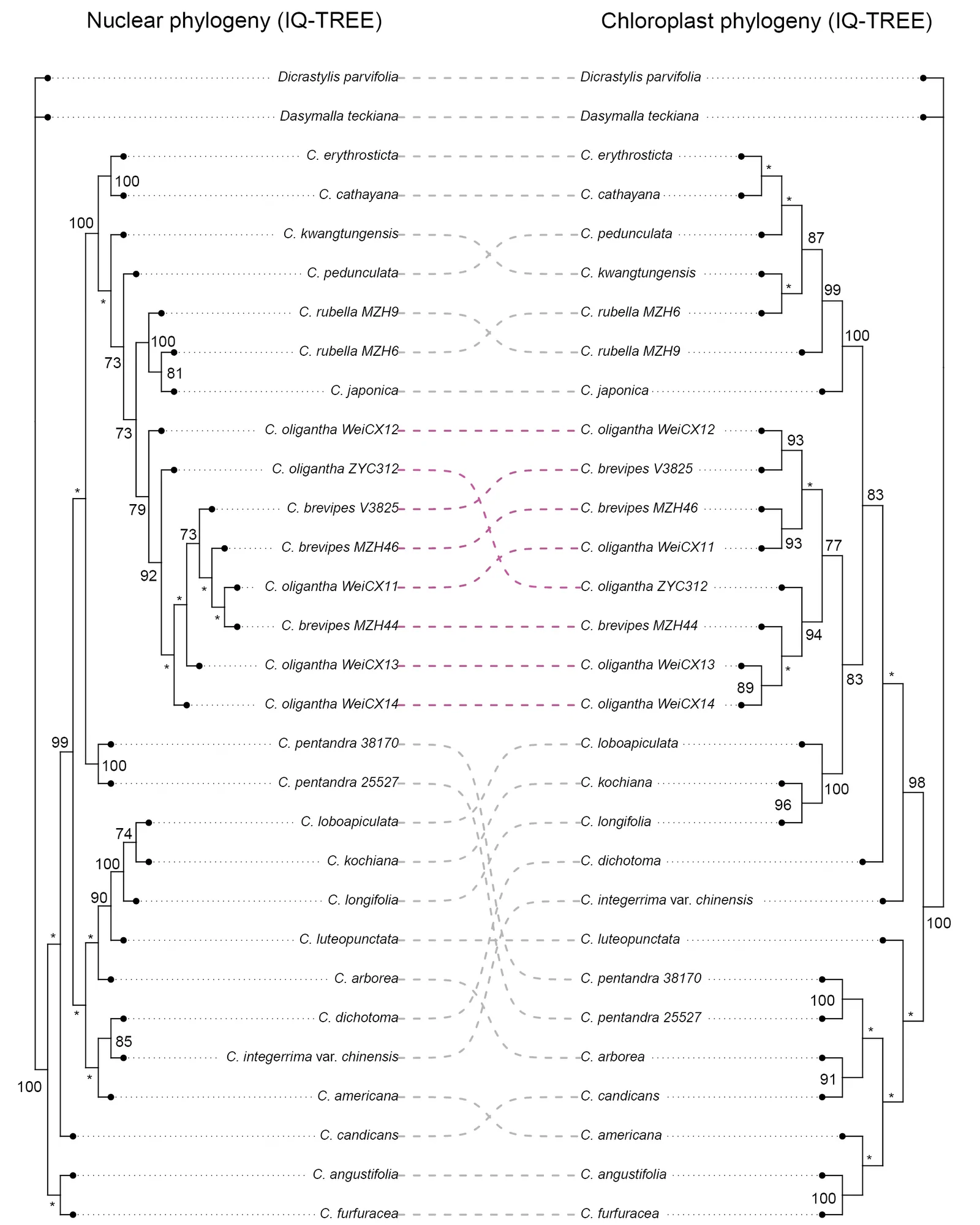
Summarized phylogenies of *Callicarpa* showing discordances between nuclear and plastid topologies. Numbers at internal branches show support values; asterisks indicate support values below 70%.

**Figure 5.**
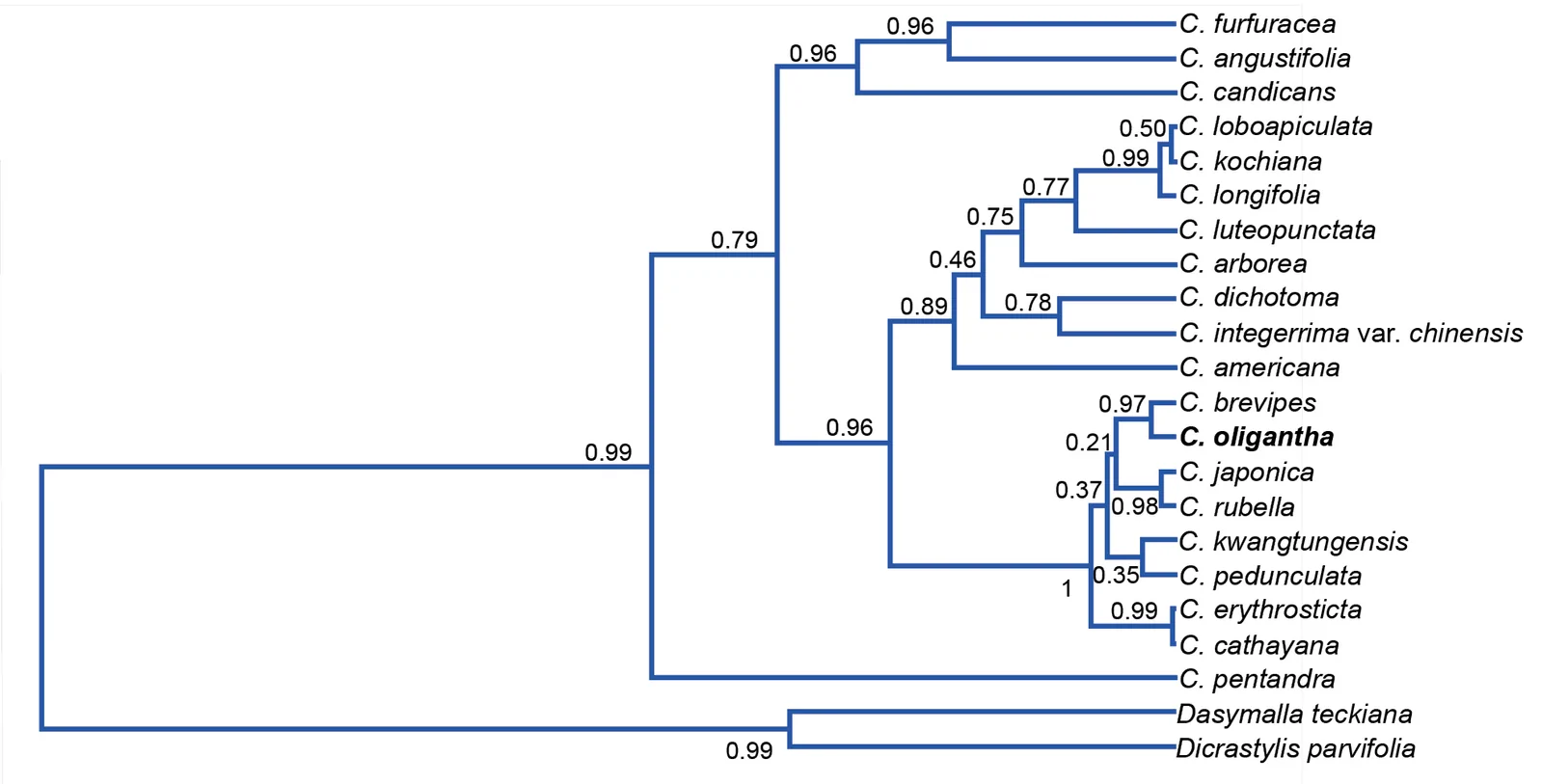
Species tree generated using StarBEAST2 based on two nrDNA regions. The number above each branch represents posterior probability.

The aligned data set consisted of 30 accessions, including 21 taxa of *Callicarpa* and two outgroup species. Two concatenated nrDNA sequence was 1087 bp in length and eight cpDNA concatenated sequence's length was 7435 bp. The variable sites of each locus ranged from 51 to 200 and the parsimony informative sites ranged from 4 to 110 (Suppl. material 1: table S5). Phylogenetic trees independently inferred for the nrDNA and cpDNA datasets using ML and BI approaches. For both nuclear and plastid phylogenies, only the ML tree is presented below, with posterior probabilities from BI analyses indicated, and nodes with conflicting topologies marked with asterisks (Figs 2, 3). Our results showed that *Callicarpa* was monophyletic (BI posterior probability = 1.00, ML bootstrap = 100). *C. oligantha* was intermingled with *C. brevipes* in both datasets (0.93/79; 0.99/77) and discordance between nuclear and plastid topologies summarized in Fig. 4, which did not affect their recovery as a monophyletic group. In other words, *C. oligantha* was distinct from other *Callicarpa* species but indistinguishable from *C. brevipes* phylogenetically, implying that *C. oligantha* does not represent an evolutionarily independent lineage. Additionally, the species trees based on the multi-species coalescent approach in both datasets respectively indicated that *C. oligantha* was sister to *C. brevipes* (0.97/0.97; Fig. 5, Suppl. material 1: fig. S1).

### Morphological analyses between *Callicarpa oligantha* and *C. brevipes*

**Figure 6.**
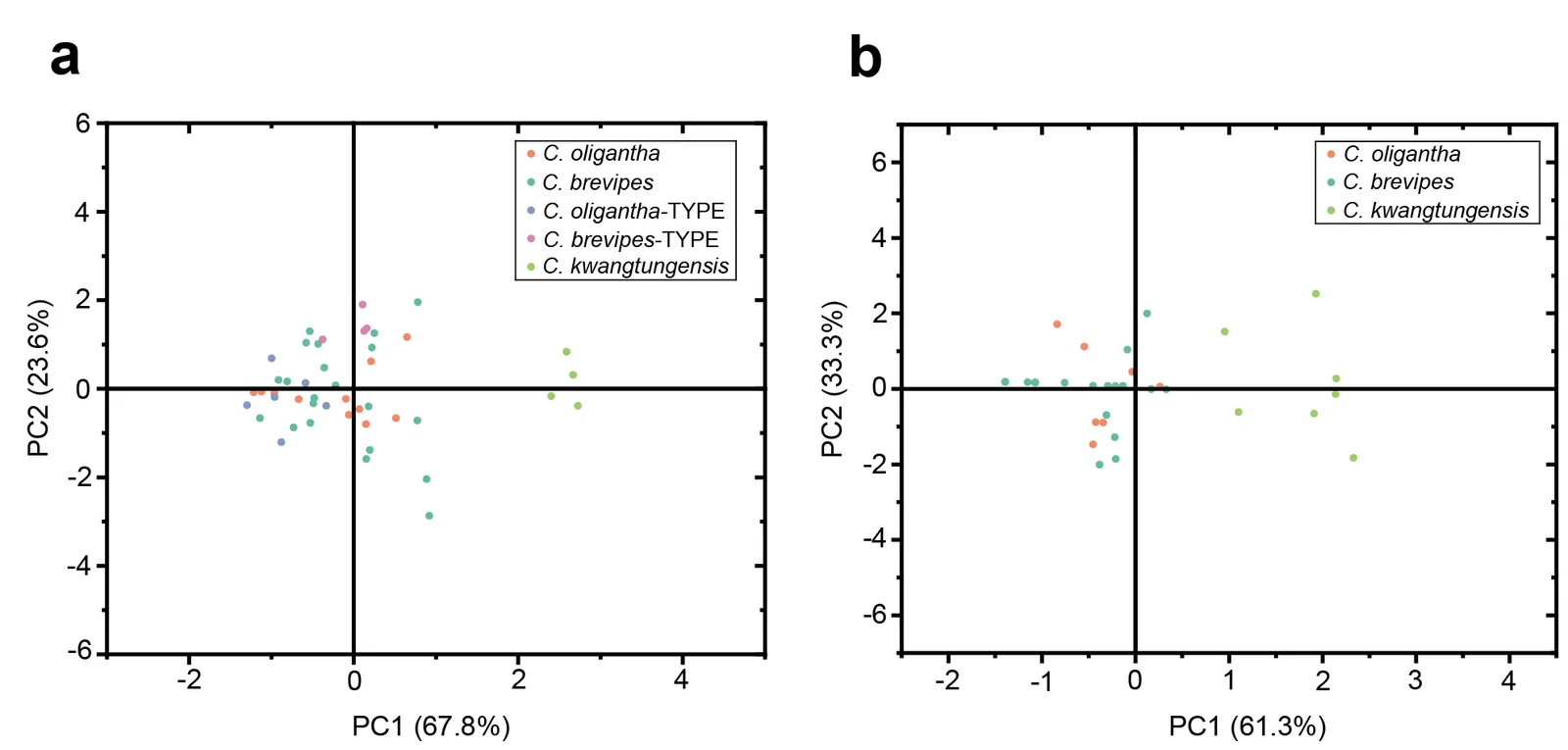
Morphological analyses of *Callicarpa oligantha* and *C. brevipes*. **a**. Scatter plot of the first two principal components (PCs) of PCA based on three leaf morphological variables of *C. oligantha* collected in this study (*C. oligantha*), *C. brevipes*, *C. oligantha*-TYPE (*C. oligantha* isotype), *C. brevipes*-TYPE (*C. brevipes* lectotype) and *C. kwangtungensis*; **b**. Scatter plot of the PCA based on three flower morphological variables of *C. oligantha*, *C. brevipes* and *C. kwangtungensis*. Each dot represents a morphometric measurement.

**Figure 7.**
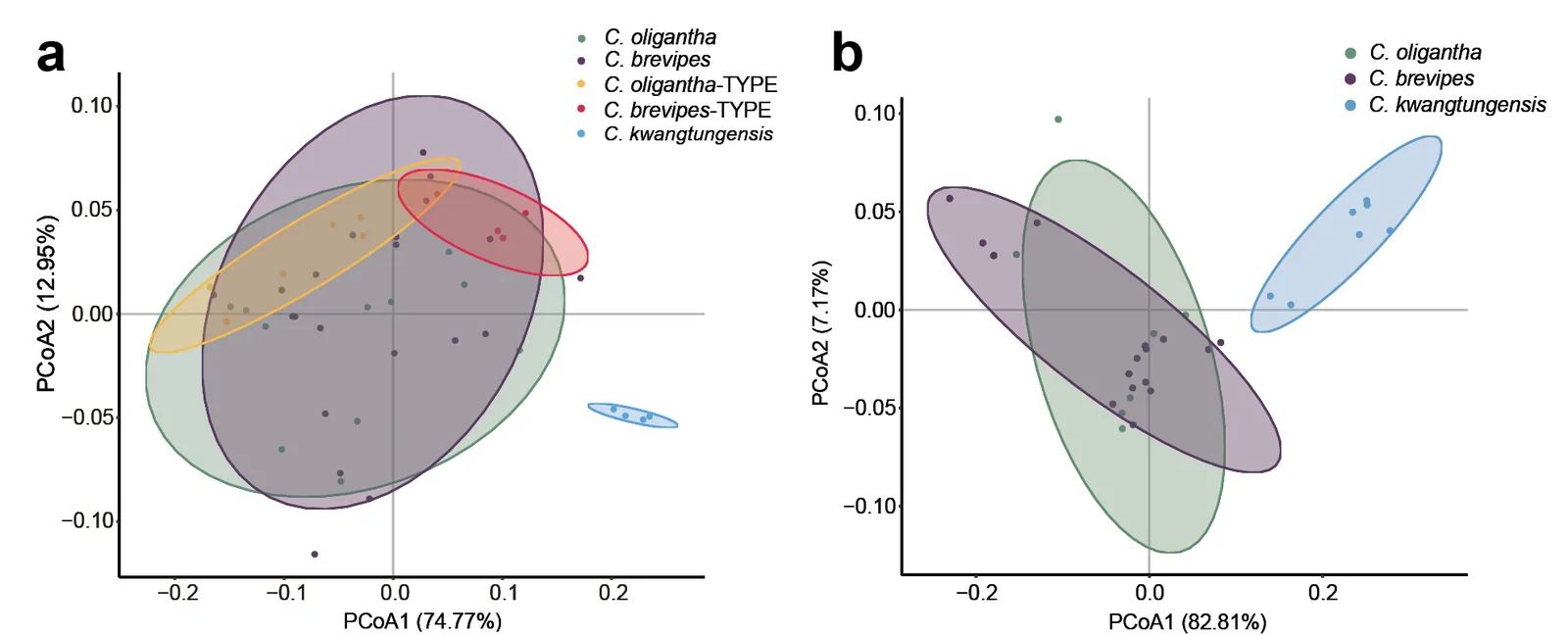
Morphological analyses of *Callicarpa oligantha* and *C. brevipes*. **a**. Scatter plot of the first two dimensions of PCoA based on three leaf morphological variables of *C. oligantha* collected in this study (*C. oligantha*), *C. brevipes*, *C. oligantha*-TYPE (*C. oligantha* isotype), *C. brevipes*-TYPE (*C. brevipes* lectotype) and *C. kwangtungensis*; **b**. Scatter plot of the PCoA based on three flower morphological variables of *C. oligantha*, *C. brevipes* and *C. kwangtungensis*. Each dot represents a morphometric measurement.

**Figure 8.**
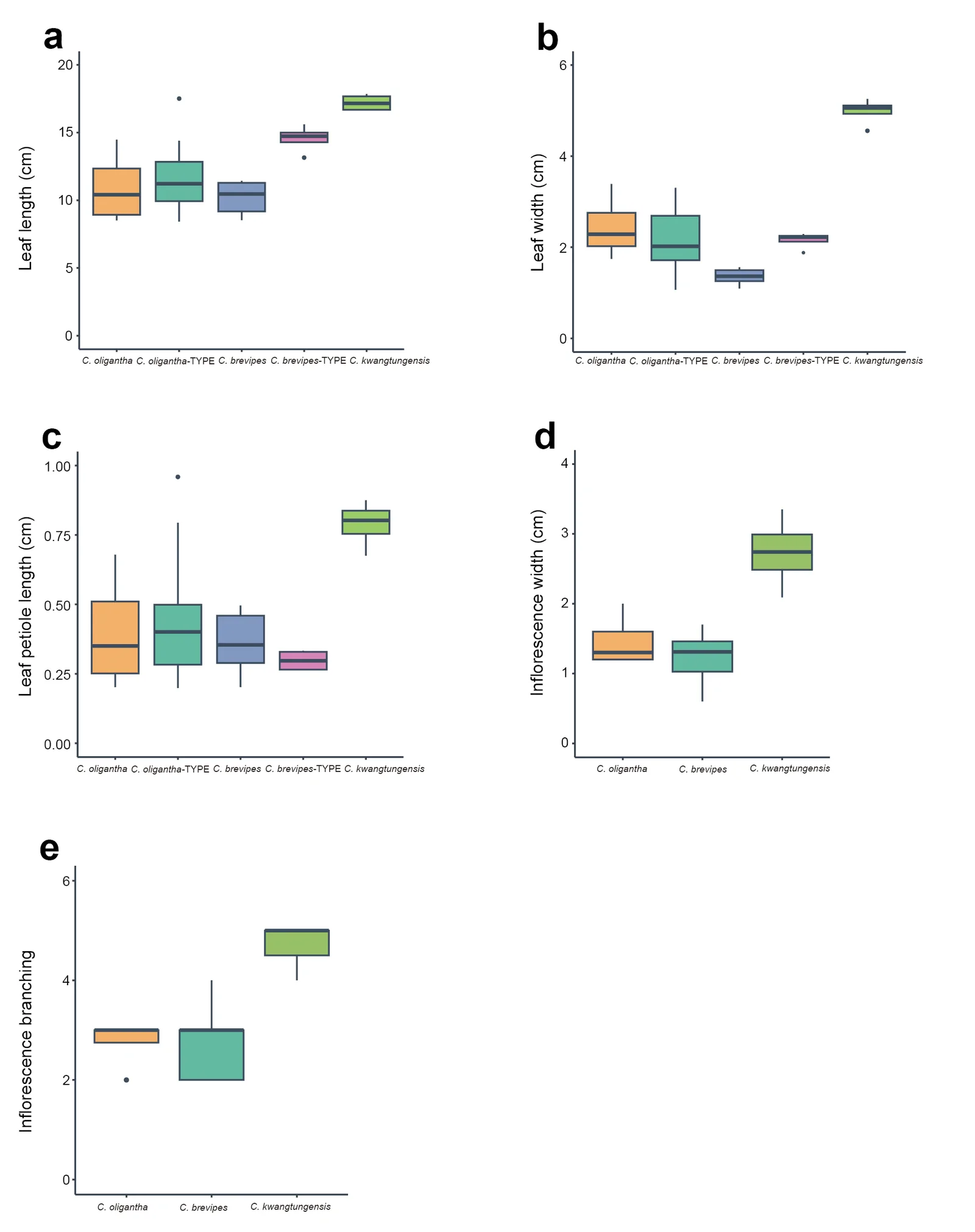
Comparisons of five morphological characters among *Callicarpa oligantha*, *C. brevipes* and *C. kwangtungensis*. **a–c**. *T*-test analyses of three leaf morphological characters among *C. oligantha*, *C. brevipes*, *C. oligantha*-TYPE (*C. oligantha* isotype), *C. brevipes*-TYPE (*C. brevipes* lectotype) and *C. kwangtungensis*. **d, e**. Two flower morphological variables of *C. oligantha*, *C. brevipes* and *C. kwangtungensis*. The boxes represent the interquartile and the vertical lines represent the range excluding the extreme values (black dots). **a**. Leaf length; **b**. Leaf width; **c**. Leaf petiole length; **d**. Inflorescence width; **e**. Inflorescence branching.

The results of PCA and PCoA were determined by the six variables (leaf length, leaf width, leaf petiole length, inflorescence width, inflorescence branching and filament length). Both PCA and PCoA clearly showed that *Callicarpa oligantha* and *C. brevipes* formed one group. In the PCA of leaf morphology, the first two axes explained 91.4% of the variance, with 67.8% for PC1 and 23.6% for PC2, respectively (Fig. 6a). Likewise, in flower morphology, the first two axes explained 94.6% of the variance, with 61.3% for PC1 and 33.3% for PC2, respectively (Fig. 6b). In PCoA of leaf morphology, the first two principal coordinates accounted for 87.72% of the variance, with 74.77% for PCoA1 and 12.95% for PCoA2, respectively (Fig. 7a). Correspondingly, in flower morphology, the first two principal coordinates accounted for 89.98% of the variance, with 82.81% for PCoA1 and 7.17% for PCoA2, respectively (Fig. 7b). When *C. oligantha* and *C. brevipes* were pairwise compared, only *C. brevipes*-TYPE showed a slight difference with other group about leaf morphology in Adonis analysis (Suppl. material 1: tables S6, S7). *T*-test analyses of six morphological characters showed that there was no significant difference between *C. oligantha* and *C. brevipes* (Fig. 8; Suppl. material 1: tables S8, S9). Notably, given that filament lengths did not differ significantly among the three species (all belonging to Ser. *Verticirimae* in *Callicarpa*), and thus were omitted from the Fig. 8 (see Suppl. material 1: table S9 for detailed values). All the original measurements of morphological traits are presented in Suppl. material 1: tables S3, S4.

## Discussion

### Taxonomic status of *Callicarpa oligantha*

In this study, *Callicarpa oligantha* and *C. brevipes* are nested together in our newly reconstructed phylogenetic tree (Figs 2, 3, 4, 5). Both nuclear and chloroplast genes clearly supported that all samples of *C. oligantha* are embedded within *C. brevipes* and meet the criteria of the phylogenetic species concept. The phylogenetic species concept has been increasingly recognized as a valuable approach for species delimitation, especially in groups where closely related species exhibit limited morphological differentiation (Lin et al. 2022, 2024). Basically, *C. oligantha* is a part of *C. brevipes* variability. In our nuclear result, the group of *C. oligantha* and *C. brevipes* was sister to a subclade containing of *C. rubella* Lindl. and *C. japonica* Thunb. In contrast, the chloroplast phylogeny recovered the clade comprising *C. oligantha* and *C. brevipes* as sister to a subclade consisting of *C. loboapiculata* Metcalf, *C. kochiana* Makino and *C. longifolia* Lam., species which have white fruit morphology (Fig. 4). However, the discordance between nuclear and plastid topologies did not affect the recovery of *C. oligantha* and *C. brevipes* as a well-supported monophyletic group (0.93/79; 0.99/77), and they represent an independent evolutionary lineage from their sister subclade. Notably, topological incongruence between the two datasets suggests a complex history involving both hybridization and incomplete lineage sorting in *Callicarpa*, which are also common biological processes in major angiosperm lineages (Huang et al. 2025; Kazutoshi et al. 2025). The PCA, PCoA and the *t*-test of morphological characters collectively demonstrated that there is little differentiation between the two species (Figs 6, 7, 8). Note that although the Adonis analysis showed a slight difference between *C. brevipes*-TYPE and other groups in leaf morphology, it showed no significant differences between *C. oligantha* and *C. brevipes* collected in this study or between *C. oligantha* collected in this study and *C. oligantha* types (Suppl. material 1: table S6). This pattern is reasonable because the fragmented distribution of *C. brevipes* may drive intraspecific morphological variation (Gao et al. 2021; Li et al. 2021, 2023). In the present study, we included the isotype specimen of *C. oligantha* specifically, a practice that may avoid the bias introduced by potential misidentification of the new *C. oligantha* collections. The first type of the species collected by Elmer Drew Merrill (No. 11060) in thickets along small streams, at an altitude of about 900 meters, Luofu Mountain, Guangdong, China, on August 23, 1917, was deposited in the herbarium of the Philippine Bureau of Science at Manila (https://www.biodiversitylibrary.org/item/47413) (Merrill 1918). Notably, the distribution of *C. oligantha* completely overlaps with that of *C. brevipes*, thus it is possible that *C. oligantha* represents a distinct population of *C. brevipes* and is a part of *C. brevipes* variability. Our comprehensive analyses strongly support that *C. oligantha* is highly likely to be conspecific with *C. brevipes* rather than an independent species, thereby resolving a nearly 110-year-old enigma in the taxonomy of *Callicarpa*.

**Figure 9.**
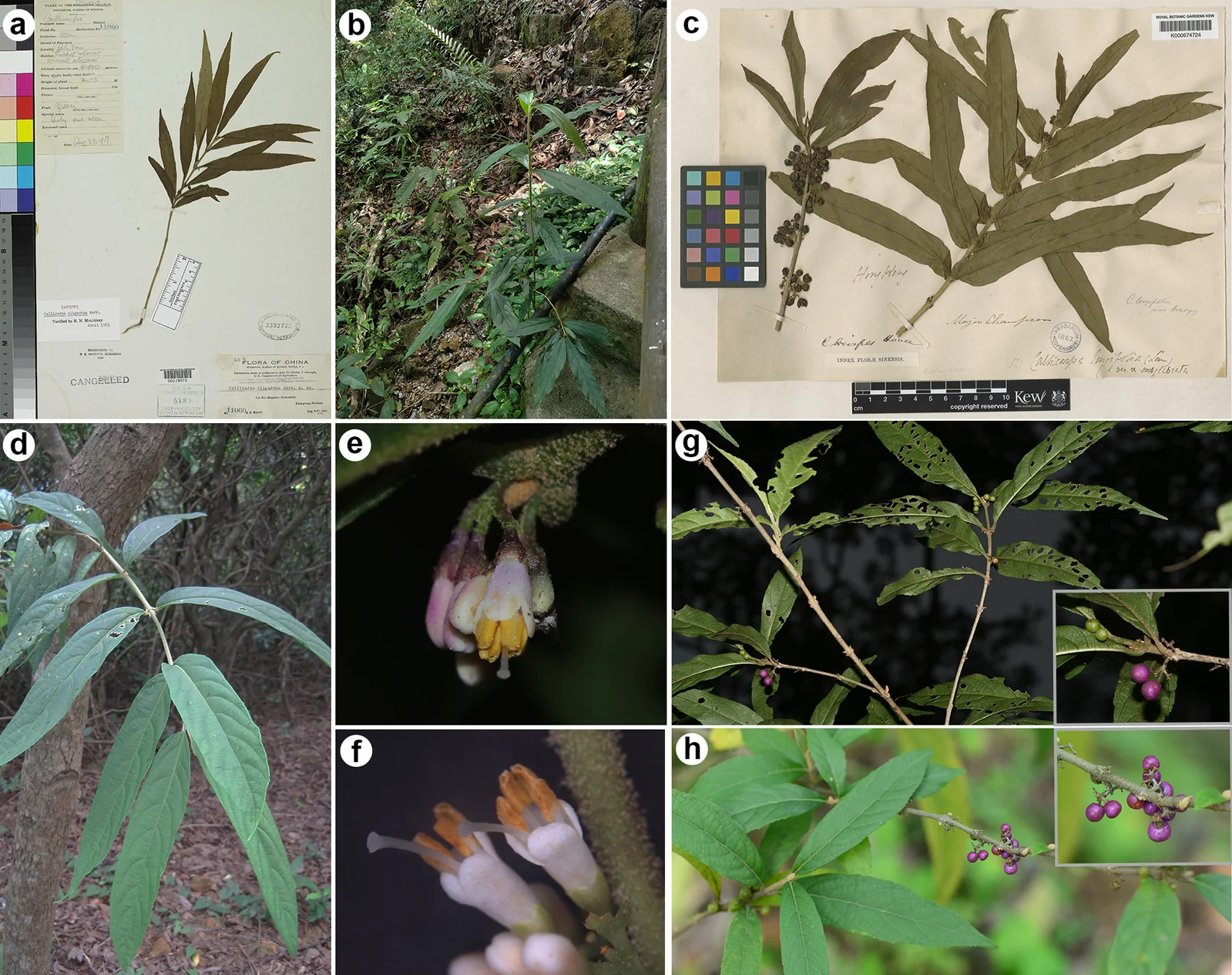
Specimens and field images of *Callicarpa oligantha* and *C. brevipes*. **a**. Isotype of *C. oligantha* deposited in the US; **b**. Field images of *C. oligantha* taken in Luofu Mountain, Guangdong, China; **c**. Lectotype of *C. brevipes* deposited in the K; **d**. Field images of *C. brevipes*; **e**. Flowers of *C. oligantha*; **f**. Flowers of *C. brevipes*; **g**. Fruits of *C. oligantha*; **h**. Fruits of *C. brevipes*.

Morphological distinctions between *Callicarpa oligantha* and *C. brevipes* described in *Flora Reipublicae Popularis Sinicae* are as follows (Wu and Li 1977): the former has cuneate leaf base with sparsely pubescent abaxial surfaces and cymes 2–3 flowered, whereas the latter exhibits obtuse leaf bases (sometimes varying from cuneate to subcordate) with pubescent abaxial surfaces and cymes 2–3 branched. However, no clear discontinuities exist in the morphological variation and they do not warrant a species split (Fig. 9; Suppl. material 1: figs S2, S3). Additionally, there are no discernible differences in the two species' leaf length (8.5–14.5 cm in *C. oligantha* vs. 8.4–17.5 cm in *C. brevipes*), leaf width (1.1–3.4 cm in *C. oligantha* vs. 1.1–3.3 cm in *C. brevipes*), the length of leaf petiole (0.2–0.7 cm in *C. oligantha* vs. 0.2–1.0 cm in *C. brevipes*), inflorescence width (1.2–2.0 cm in *C. oligantha* vs. 0.6–1.7 cm in *C. brevipes*), inflorescence branching (2–3 in *C. oligantha* vs. 2–4 in *C. brevipes*) and filament length (0.22–0.38 cm in *C. oligantha* vs. 0.19–0.4 cm in *C. brevipes*). And the leaf blades of both are lanceolate to narrowly lanceolate. Based on the high similarities in leaf blade shape and size of leaf blade, we also reasonably suggested that *C. oligantha* indeed is conspecific with *C. brevipes*.

### Conservation implications

The absence of a stable taxonomic framework impedes the formulation of effective conservation strategies to protect biodiversity (Garnett and Christidis 2017; Sun et al. 2017; Thomson et al. 2018). A case in point is the clover fern *Marsilea azorica* Launert & Paiva. It was listed as a conservation priority species in the Azores, Macaronesia and Europe, and the population was managed by the local conservation agency, classified as 'critically endangered' on the IUCN red list, and as 'strictly protected' species by the Bern convention and the European Union's habitats directive (Schaefer et al. 2011). However, its taxonomy and phylogenetic relationships had been largely neglected. Schaefer et al. (2011) combined morphological and molecular data (*rbcL* gene, *rps4* gene, *rps4*-*trnS* spacer and *trnL*-*trnF* spacer sequences) and demonstrated that *M. azorica* was conspecific with *M. hirsute* R.Br., which is an invasive plant and advised that it should be clearly removed from all conservation priority lists and the IUCN red list. It follows that even the most sophisticated conservation priority ranking schemes would fail without a solid taxonomic basis. To avoid directing precious conservation resources to misidentified species, a comprehensive and detailed reassessment of the taxonomy is needed. As no additional individuals of "*Callicarpa oligantha*" had been found since its first discovery, some researchers deservedly believed that this species was extremely endangered or on the edge of extinction (Wang 2022). Another species assessed as probably extinct in Guangdong is *Corsiopsis chinensis* D. X. Zhang, R. M. K. Saunders & C. M. Hu, which has also not been observed during field surveys for many years. An unusual herbarium specimen of *C. chinensis* was collected in Guangdong, China (*Yue-74 Expedition 5072*, IBSC) and it was described as a new species in 1999, representing the first record of *C. chinensis* in Asia (Zhang et al. 1999). Accordingly, the local forestry authorities are likely to include “*C. oligantha*” in the list of key protected wild plants, and substantial conservation efforts will be undertaken to protect this rare species. In this study, however, we demonstrated that "*C. oligantha*" is conspecific with *C. brevipes* and the name should be placed in synonomy under *C. brevipes* following the International Code of Nomenclature for Algae, Fungi, and Plants (Shenzhen Code): Article 11 (Turland et al. 2018), which is a widely distributed species. According to both the IUCN and the Red List of China's Biodiversity: Higher Plants (2020) (Ministry of Ecology and Environment of the People's Republic of China, and Chinese Academy of Sciences 2023), this species is assessed as 'least concern' due to its stable population and absence of major threats. Specifically, to further reveal patterns of intraspecific genetic diversity of *C. brevipes*, population genomic studies with expanded sampling across its entire distribution range is necessary. Therefore, *C. oligantha* should not be considered as extinct. This taxonomic conclusion will enable better prioritization of conservation planning and management allowing resources to be focused away from widespread Least Concern species and towards managing real threats to plant diversity.

## Taxonomic treatment

### Callicarpa brevipes

Taxon classificationPlantaeLamialesLamiaceae

(Benth.) Hance in Ann. Sci. Nat. 5(5): 233. 1886

08233792-8195-5B35-838B-6A435494F302

≡ C. longifolia var. brevipes Benth. Fl. Hongk. 270, 1861 – Type: China, Hongkong: *Champion s.n*. (**lectotype, designated here**: K barcode K000674724!).
= C. oligantha Merr. in Philip. Journ. Sci. Bot. 13: 155. 1918. syn. nov. – Type: China, Guangdong: Luofu Mountain, alt. *ca*. 900 m, 23 Aug. 1917, *Merrill 11060* (**holotype**, NY barcode 00103957!; **isotype**, US barcode 00119073!).

#### Description.

Shrubs *ca*. 1–2.5 m tall, slender. Branchlets terete or obscurely 4-angled, yellow-brown stellate pubescent when young, glabrescent. Leaves decussate, petiole *ca*. 0.2–1.0 cm, canaliculate above, stellate-pubescent, leaf blades bright green, nitid, lighter beneath, narrowly lanceolate or oblong, *ca*. 8.4–17.5 × 1.1–3.4 cm, acuminate or caudate-acuminate at apex, abaxially stellate pubescent on veins and yellow glandular, adaxially glabrous or with stellate hairs on the venation, serrate for the upper 1/2–2/3 of the margins, veins 9–12 pairs. inflorescence axillary, small, peduncle slender, subequal to slightly longer than petioles, peduncles densely stellate-pubescent. Flowers small, usually pendent. Calyx cup-shaped or campanulate, glabrate or sparsely stellate-pubescent, subtruncate and 4-dentate. Corolla white, *ca*. 3.5 mm, glabrous. Filaments nearly as long as corolla; anthers oblong, densely yellow glandular. Ovary with stellate tomentose and yellow glandular. Style longer than stamens. Fruits purple.

#### Phenology.

It starts to flower from April to June; its fruits are mature from July to November.

#### Distribution and habitat.

It is mainly known in Southeast China (Guangdong, Guangxi, Hainan and Hongkong), and Vietnam, usually growing along streams or in open forests between 600–1400 m.

#### Note.

The first valid publication of the name *Callicarpa brevipes* (Benth.) Hance appeared in the Annales des Sciences Naturelles by Hance in 1866 (Hance 1866). The history of this species’ recognition is complex. In 1825, Hooker described a species of *Callicarpa* based on a living plant from China, which he thought that it represented *Callicarpa longifolia* Lamarck (Hooker 1825). However, Bentham (1861) recognized that the plant Hooker described was not Lamarck’s species (*Callicarpa longifolia*) and treated it as a variety Callicarpa longifolia var. brevipes Benth (Bentham 1861). Subsequently, Hance raised this variety to species rank as *C. brevipes* (Hance 1866).

We reviewed the protologue of *Callicarpa brevipes* to determine if any original material representing this species has been cited. Bentham (1861) referred three collectors Champion, Wright and Wilford but did not explicitly designate a type specimen. No subsequent lectotype was designated by anyone, except for the direct assignment of a specimen collected by Champion as the holotype (*Champion s.n*., K barcode K000674724) by Ma (2013) without providing any justification. Hence, we further checked key herbarium for Champion, Wright and Wilford's collections. There is a specimen of Champion (*Champion s.n*., K barcode K000674724) in the Kew Herbarium, which matches the description in the protologue and is preserved well with leaves, flowers and fruits. Therefore, we designed this specimen as a lectotype.

## Supplementary Material

XML Treatment for Callicarpa brevipes

## Additional information

### Artificial Intelligence (AI) use

The authors accept full responsibility for the content of the manuscript, including the disclosure of any use of AI.

No AI tools were used in the preparation of this manuscript.

### Author ORCIDs

H. Cai https://orcid.org/0000-0003-0647-8219

Q. Yang https://orcid.org/0000-0003-4835-1558

T. Tu https://orcid.org/0000-0001-7385-008X

Z. Ma https://orcid.org/0000-0002-3898-3079

### Data availability

The datasets generated during the current study were deposited in GenBank (http://www.ncbi.nlm.nih.gov) and the accession numbers were shown in Table 2.

## Supplementary materials

Supplementary material 1 Suppl. tables and figures about phylogenetic and morphological data Data type docx Explanation note **table SS1**. Primers used to amplify and sequence nrDNA and cpDNA in this study. **table SS2**. The best-fitting nucleotide substitution models selected for BI and ML analyses. **table S3**. Original leaf morphology measurements in this study. **table S4**. Original flower morphology measurements in this study. **table S5**. Site information of two nrDNA loci and eight cpDNA loci in this study. **table S6**. Pairwise comparisons of *Callicarpa oligantha* collected in this study (*C. oligantha*), *C. brevipes*, *C. oligantha*-TYPE (*C. oligantha* isotype), *C. brevipes*-TYPE (*C. brevipes* lectotype) and *C. kwangtungensis* based on three leaf morphological characters in Adonis analysis using pairwise Adonis package. **table S7**. Pairwise comparisons of *Callicarpa oligantha*, *C. brevipes* and *C. kwangtungensis* collected in this study based on three flower morphological characters in Adonis analysis using pairwise Adonis package. **table S8**. T-test analyses of three leaf morphological characters among *Callicarpa oligantha*, *C. brevipes*, *C. oligantha*-TYPE (*C. oligantha* isotype), *C. brevipes*-TYPE (*C. brevipes* lectotype) and *C. kwangtungensis*. **table S9**. T-test analyses of three flower morphological characters among *Callicarpa oligantha*, *C. brevipes* and *C. kwangtungensis*. **fig. S1**. Species tree generated using StarBEAST2 based on eight cpDNA regions. **fig. S2**. Specimens of *Callicarpa oligantha* and *C. brevipes* and all specimens are deposited in the herbarium of the College of Agriculture, Guangxi University (GAUA). **fig. S3**. Stellate indumentum of *Callicarpa oligantha* and *C. brevipes* on leaves and fruits. https://binary.pensoft.net/file/1747210This dataset is made available under the Open Database License (http://opendatacommons.org/licenses/odbl/1.0/). The Open Database License (ODbL) is a license agreement intended to allow users to freely share, modify, and use this Dataset while maintaining this same freedom for others, provided that the original source and author(s) are credited. Huimin Cai, Qiyi Yang, Yicheng Xu, Tieyao Tu, Zhonghui Ma
