# Supplementary material for: Rediscovery and reexamination of a declared 'extinct' plant in South China: The species delimitation and conservation implications of Callicarpa oligantha (Lamiaceae)
Source: PhytoKeys. 2026 Aug 17;278:157–76. doi: 10.3897/phytokeys.278.203604 (PMC13504310; doi:10.3897/phytokeys.278.203604)
Supplement: Supplementary file 1 [file phytokeys-278-157_article-203604__-s001.docx]

**Rediscovery and reexamination of a declared 'extinct' plant in South China: the species delimitation and conservation implications of *Callicarpa oligantha* (Lamiaceae)**

Huimin Cai^1^, Qiyi Yang^2^, Yicheng Xu^3^, Tieyao Tu^2,^ *, Zhonghui Ma^1,^ *

^1^National Demonstration Center for Experimental Plant Science Education, College of Agriculture, Guangxi University, Nanning 530004, Guangxi, China

^2^State Key Laboratory of Plant Diversity and Specialty Crops / Guangdong Provincial Key Laboratory of Applied Botany, South China Botanical Garden, Chinese Academy of Sciences, Guangzhou 510650, China

^3^Luofu Mountain State-owned Forest Farm, Huizhou 516100, China

Tieyao Tu: [tutieyao@scbg.ac.cn](mailto:tutieyao@scbg.ac.cn) (* corresponding authors)

Zhonghui Ma: [mazhonghui@gxu.edu.cn](mailto:mazhonghui@gxu.edu.cn) (* corresponding authors)

**Suppl. Tables and Figures List**

**Table S1.** Primers used to amplify and sequence nrDNA and cpDNA in this study.

**Table S2.** The best-fitting nucleotide substitution models selected for BI and ML analyses.

**Table S3.** Original leaf morphology measurements in this study.

**Table S4.** Original flower morphology measurements in this study.

**Table S5.** Site information of two nrDNA loci and eight cpDNA loci in this study.

**Table S6.** Pairwise comparisons of *Callicarpa oligantha* collected in this study (*C. oligantha*), *C. brevipes*, *C. oligantha*-TYPE (*C. oligantha* isotype), *C. brevipes*-TYPE (*C. brevipes* lectotype) and *C. kwangtungensis* based on three leaf morphological characters in Adonis analysis using pairwise Adonis package.

**Table S7.** Pairwise comparisons of *Callicarpa oligantha*, *C. brevipes* and *C. kwangtungensis* collected in this study based on three flower morphological characters in Adonis analysis using pairwise Adonis package.

**Table S8.** *T*-test analyses of three leaf morphological characters among *Callicarpa oligantha*, *C. brevipes*, *C. oligantha*-TYPE (*C. oligantha* isotype), *C. brevipes*-TYPE (*C. brevipes* lectotype) and *C. kwangtungensis*.

**Table S9.** *T*-test analyses of three flower morphological characters among *Callicarpa oligantha*, *C. brevipes* and *C. kwangtungensis*.

**Fig. S1** Species tree generated using StarBEAST2 based on eight cpDNA regions. The number above each branch represents posterior probability.

**Fig. S2** Specimens of *Callicarpa oligantha* and *C. brevipes* and all specimens are deposited in the herbarium of the College of Agriculture, Guangxi University (GAUA). a. *C. oligantha* (No. WeiCX11); b. *C. oligantha* (No. ZYC312); c. *C. oligantha* (No. WeiCX14); d. *C. oligantha* (No. WeiCX13); e. *C. oligantha* (No. WeiCX12); f. *C. brevipes* (No. 6); g. *C. brevipes* (No. 078); h. *C. brevipes* (No. 082); i. *C. brevipes* (No. 0150); j. *C. brevipes* (No. LB0169).

**Fig. S3** Stellate indumentum of *Callicarpa oligantha* and *C. brevipes* on leaves and fruits*.* a-b. Adaxial leaf surface of *C. oligantha*. c-d. Abaxial leaf surface of *C. oligantha*. e. fruits of *C. oligantha*. f-g. Adaxial leaf surface of *C. brevipes*. h-i. Abaxial leaf surface of *C. brevipes*. j. fruits of *C. brevipes*.

| **Table S1 Primers used to amplify and sequence nrDNA and cpDNA in this study.** | | | |
| --- | --- | --- | --- |
| **Marker** | **Primer** | **Sequence (5’ to 3’)** | **Reference** |
| ITS | 17SE | ACGAATTCATGGTCCGGTGAAGTGTTCG | Sun et al. 1994 |
|  | 26SE | TAGAATTCCCCGGTTCGCTCGCCGTTAC |  |
|  | ITS-4 | TCCTCCGCTTATTGATATGC | White et al. 1990 |
|  | ITS-5 | GGAAGTAAAAGTCGTAACAAGG |  |
| ETS | 18S-IGS | GAGACAAGCATATGACTACTGGCAGGATCAACCAG | Baldwin and Markos 1998 |
|  | ETS-B | ATAGAGCGCGTGAGTGGTG | Beardsley and Olmstead 2002 |
| *matK* | 323f | ATTNTCAAATCNTAKCAGAGGGG | Andersson 2006 |
|  | 1189r | CGGCTTACTAATRGGATGCCC |  |
| *psbJ*-*petA* | psbJ | ATAGGTACTGTARCYGGTATT | Shaw et al. 2007 |
|  | petA | AACARTTYGARAAGGTTCAATT |  |
|  | psbJF1 | TAGGTTCATCCCTGTAGT | Designed here |
|  | petAR1 | TTTTGTTGTCAATTATGT |  |
| *rpL32*-*trnL* | trnL(UAG) | CTGCTTCCTAAGAGCAGCGT | Shaw et al. 2007 |
|  | rpL32-F | CAGTTCCAAAAAAACGTACTTC |  |
| *trnD*-*trnT* | trnDF | ACCAATTGAACTACAATCCC | Demesure et al. 1995 |
|  | trnT | CTACCACTGAGTTAAAAGGG |  |
| *trnS (GCU)-trnG* | trnS (GCU) | AGATAGGGATTCGAACCCTCGGT | Shaw et al. 2005 |
|  | 5’trnG2S | TTTTACCACTAAACTATACCCGC |  |
| *trnH*-*psbA* | psbAF | GTTATGCATGAACGTAATGCTC | Sang et al. 1997 |
|  | trnH2 | CGCGCATGGTGGATTCACAATCC | Tate and Simpson 2003 |
| *trnQ*-5'*rps16* | trnQ(UUG) | GCGTGGCCAAGYGGTAAGGC | Shaw et al. 2007 |
|  | rps16x1 | GTTGCTTTYTACCACATCGTTT |  |
| 3'*trnV*-*ndhC* | trnV(UAC) x2 | GTCTACGGTTCGARTCCGTA | Shaw et al. 2007 |
|  | ndhC | TATTATTAGAAATGYCCARAAAATATCATATTC |  |
|  | ndhC-f | AGACCATTCCAATGCCCCCTTTCGCC | Dong et al. 2012 |
|  | trnV-r | GTTCGAGTCCGTATAGCCCTA |  |

**Table S2.** The best-fitting nucleotide substitution models selected for BI and ML analyses.

| **ID** | **BI model** | **ML model** |
| --- | --- | --- |
| ITS | HKY+F+G4 | TIM2+F+G4 |
| ETS | GTR+F+G4 | TIM2+F+G4 |
| *matK* | HKY+F+G4 | K3Pu+F+G4 |
| *rpl32-trnL* | GTR+F+G4 | K3Pu+F+G4 |
| *trnD-trnT* | HKY+F+G4 | K3Pu+F+G4 |
| *trnH-psbA* | F81+F+G4 | F81+F+G4 |
| *psbJ-petA* | HKY+F+G4 | K3Pu+F+G4 |
| *trnQ-5’rps16* | GTR+F+G4 | K3Pu+F+G4 |
| *3’trnV-ndhC* | HKY+F+G4 | K3Pu+F+G4 |
| *trnS (GCU)-trnG* | HKY+F+G4 | K3Pu+F+G4 |

**Table S3.** Original leaf morphology measurements in this study.

| **Species** | **Voucher ID (collection number)** | **Herbarium barcode** | **Collector** | **Leaf length (cm)** | **Leaf width (cm)** | **Leaf petiole length (cm)** |
| --- | --- | --- | --- | --- | --- | --- |
| *C. oligantha* | WeiCX11 | ─ | Wei et al. | 9.871 | 3.244 | 0.336 |
| *C. oligantha* | WeiCX11 | ─ | Wei et al. | 8.773 | 2.645 | 0.257 |
| *C. oligantha* | WeiCX11 | ─ | Wei et al. | 10.410 | 2.867 | 0.510 |
| *C. oligantha* | ZYC312 | ─ | Zhang et al. | 14.482 | 3.391 | 0.350 |
| *C. oligantha* | WeiCX14 | ─ | Wei et al. | 13.435 | 2.514 | 0.402 |
| *C. oligantha* | WeiCX13 | ─ | Wei et al. | 13.121 | 2.096 | 0.679 |
| *C. oligantha* | WeiCX13 | ─ | Wei et al. | 11.080 | 2.186 | 0.511 |
| *C. oligantha* | WeiCX13 | ─ | Wei et al. | 11.584 | 2.286 | 0.517 |
| *C. oligantha* | WeiCX12 | ─ | Wei et al. | 9.051 | 1.948 | 0.246 |
| *C. oligantha* | WeiCX12 | ─ | Wei et al. | 8.816 | 1.776 | 0.221 |
| *C. oligantha* | WeiCX12 | ─ | Wei et al. | 8.508 | 1.744 | 0.202 |
| *C. brevipes* | 6 | ─ | Ma ZH | 10.379 | 1.717 | 0.283 |
| *C. brevipes* | 6 | ─ | Ma ZH | 12.039 | 1.888 | 0.426 |
| *C. brevipes* | 6 | ─ | Ma ZH | 8.427 | 1.373 | 0.333 |
| *C. brevipes* | 078 | ─ | Ma ZH | 9.092 | 3.219 | 0.548 |
| *C. brevipes* | 078 | ─ | Ma ZH | 10.338 | 3.306 | 0.794 |
| *C. brevipes* | 078 | ─ | Ma ZH | 9.935 | 2.786 | 0.959 |
| *C. brevipes* | 082 | ─ | Ma ZH | 9.663 | 1.979 | 0.221 |
| *C. brevipes* | 082 | ─ | Ma ZH | 11.227 | 1.531 | 0.434 |
| *C. brevipes* | 082 | ─ | Ma ZH | 10.012 | 1.067 | 0.499 |
| *C. brevipes* | 0150 | ─ | Ma ZH | 9.780 | 1.775 | 0.458 |
| *C. brevipes* | 0150 | ─ | Ma ZH | 14.401 | 2.241 | 0.401 |
| *C. brevipes* | 0150 | ─ | Ma ZH | 10.079 | 2.121 | 0.376 |
| *C. brevipes* | LB0169 | ─ | Li B | 13.651 | 3.122 | 0.267 |
| *C. brevipes* | LB0169 | ─ | Li B | 17.509 | 2.549 | 0.391 |
| *C. brevipes* | LB0169 | ─ | Li B | 12.929 | 1.498 | 0.267 |
| *C. brevipes* | 12 | K000674726 | Champion | 13.053 | 1.712 | 0.283 |
| *C. brevipes* | 12 | K000674726 | Champion | 12.839 | 1.922 | 0.199 |
| *C. brevipes* | 12 | K000674726 | Champion | 11.986 | 2.020 | 0.328 |
| *C. brevipes* | 442 | K000674725 | Bentham | 12.826 | 2.801 | 0.670 |
| **Table S3.** Original leaf morphology measurements in this study. (Continued) | | | | | | |
| **Species** | **Voucher ID (collection number)** | **Herbarium barcode** | **Collector** | **Leaf length (cm)** | **Leaf width (cm)** | **Leaf petiole length (cm)** |
| *C. brevipes* | 442 | K000674725 | Bentham | 9.801 | 2.461 | 0.650 |
| *C. brevipes* | 442 | K000674725 | Bentham | 11.428 | 2.692 | 0.492 |
| *C. oligantha-*isotype | 11060 | US00119073 | Merrill | 11.439 | 1.508 | 0.370 |
| *C. oligantha-*isotype | 11060 | US00119073 | Merrill | 10.931 | 1.468 | 0.202 |
| *C. oligantha-*isotype | 11060 | US00119073 | Merrill | 11.413 | 1.563 | 0.489 |
| *C. oligantha-*isotype | 11060 | US00119073 | Merrill | 8.532 | 1.254 | 0.273 |
| *C. oligantha-*isotype | 11060 | US00119073 | Merrill | 9.995 | 1.259 | 0.338 |
| *C. oligantha-*isotype | 11060 | US00119073 | Merrill | 8.905 | 1.094 | 0.496 |
| *C. brevipes-*lectotype | *s.n.* | K000674724 | Champion | 14.802 | 2.290 | 0.328 |
| *C. brevipes-*lectotype | *s.n.* | K000674724 | Champion | 13.152 | 1.881 | 0.266 |
| *C. brevipes-*lectotype | *s.n.* | K000674724 | Champion | 15.611 | 2.204 | 0.263 |
| *C. brevipes-*lectotype | *s.n.* | K000674724 | Champion | 14.669 | 2.237 | 0.333 |
| *C. kwangtungensis* | 0124 | ─ | Ma ZH | 17.853 | 5.257 | 0.675 |
| *C. kwangtungensis* | 0124 | ─ | Ma ZH | 16.689 | 5.063 | 0.875 |
| *C. kwangtungensis* | 0124 | ─ | Ma ZH | 17.623 | 5.053 | 0.78 |
| *C. kwangtungensis* | 0124 | ─ | Ma ZH | 16.68 | 4.557 | 0.825 |

**Table S4.** Original flower morphology measurements in this study.

| **Species** | **Voucher ID (collection number)** | **Herbarium barcode** | **Collector** | **Inflorescence width (cm)** | **Inflorescence branching** | **Filament length (cm)** |
| --- | --- | --- | --- | --- | --- | --- |
| *C. oligantha* | TUTY6070 | ─ | Tu et al. | 2.00 | 3 | 0.30 |
| *C. oligantha* | TUTY6070 | ─ | Tu et al. | 1.60 | 2 | 0.35 |
| *C. oligantha* | TUTY6071 | ─ | Tu et al. | 1.20 | 2 | 0.38 |
| *C. oligantha* | TUTY6071 | ─ | Tu et al. | 1.60 | 3 | 0.32 |
| *C. oligantha* | TUTY6071 | ─ | Tu et al. | 1.20 | 3 | 0.25 |
| *C. oligantha* | TUTY6071 | ─ | Tu et al. | 1.30 | 3 | 0.25 |
| *C. oligantha* | TUTY6071 | ─ | Tu et al. | 1.30 | 3 | 0.30 |
| *C. oligantha* | TUTY6071 | ─ | Tu et al. | 1.30 | 3 | 0.30 |
| *C. brevipes* | 6 | ─ | Ma ZH | 1.40 | 2 | 0.3 |
| *C. brevipes* | 0130 | ─ | Ma ZH | 1.00 | 2 | 0.3 |
| *C. brevipes* | 0130 | ─ | Ma ZH | 0.60 | 2 | 0.3 |
| *C. brevipes* | 0130 | ─ | Ma ZH | 0.90 | 2 | 0.3 |
| *C. brevipes* | 0130 | ─ | Ma ZH | 0.60 | 2 | 0.3 |
| *C. brevipes* | 082 | ─ | Ma ZH | 1.20 | 4 | 0.3 |
| *C. brevipes* | 082 | ─ | Ma ZH | 1.30 | 3 | 0.3 |
| *C. brevipes* | 082 | ─ | Ma ZH | 1.00 | 2 | 0.3 |
| *C. brevipes* | 078 | ─ | Ma ZH | 1.70 | 3 | 0.4 |
| *C. brevipes* | 078 | ─ | Ma ZH | 1.30 | 3 | 0.3 |
| *C. brevipes* | 078 | ─ | Ma ZH | 1.50 | 3 | 0.3 |
| *C. brevipes* | LB0169 | ─ | Li B | 1.50 | 3 | 0.35 |
| *C. brevipes* | LB0169 | ─ | Li B | 1.40 | 4 | 0.3 |
| *C. brevipes* | 0133 | ─ | Ma ZH | 1.00 | 2 | 0.3 |
| *C. brevipes* | 0133 | ─ | Ma ZH | 1.20 | 4 | 0.3 |
| *C. brevipes* | 0133 | ─ | Ma ZH | 1.10 | 3 | 0.3 |
| *C. brevipes* | 0133 | ─ | Ma ZH | 1.40 | 3 | 0.3 |
| *C. brevipes* | 0133 | ─ | Ma ZH | 1.50 | 3 | 0.3 |
| *C. brevipes* | zlx2465 | IBSC1011661 | Zhou et al. | 1.32 | 3 | 0.19 |
| *C. brevipes* | DSW3838 | IBSC1028900 | Deng and Zeng | 1.33 | 3 | 0.26 |
| *C. brevipes* | DSW3838 | IBSC1028900 | Deng and Zeng | 1.48 | 3 | 0.23 |
| *C. brevipes* | DSW3931 | IBSC1027227 | Deng SW | 1.53 | 3 | 0.20 |
| **Table S4.** Original flower morphology measurements in this study. (Continued) | | | | | | |
| **Species** | **Voucher ID (collection number)** | **Herbarium barcode** | **Collector** | **Inflorescence width (cm)** | **Inflorescence branching** | **Filament length (cm)** |
| *C. kwangtungensis* | 8671 | IBSC0005023 | Chen HY | 2.56 | 5 | 0.44 |
| *C. kwangtungensis* | 01147 | IBSC0717272 | Li MH | 2.98 | 5 | 0.32 |
| *C. kwangtungensis* | 01147 | IBSC0717272 | Li MH | 2.41 | 4 | 0.27 |
| *C. kwangtungensis* | LFS205 | IBSC0917638 | Lin et al. | 2.09 | 4 | 0.38 |
| *C. kwangtungensis* | 0124 | ─ | Ma ZH | 3.00 | 5 | 0.30 |
| *C. kwangtungensis* | 324 | ─ | Ma et al. | 3.35 | 5 | 0.21 |
| *C. kwangtungensis* | 324 | ─ | Ma et al. | 2.74 | 5 | 0.27 |

**Table S5.** Site information of two nrDNA loci and eight cpDNA loci in this study.

| **ID** | **Product size (bp)** | **Variable sites** | **Parsimony informative sites** |
| --- | --- | --- | --- |
| ITS | 646 | 192 | 60 |
| ETS | 441 | 116 | 15 |
| *matK* | 607 | 52 | 4 |
| *rpl32-trnL* | 892 | 109 | 37 |
| *trnD-trnT* | 847 | 51 | 8 |
| *trnH-psbA* | 710 | 128 | 68 |
| *psbJ-petA* | 1091 | 107 | 17 |
| *trnQ-5’rps16* | 1316 | 200 | 110 |
| *3’trnV-ndhC* | 1184 | 103 | 23 |
| *trnS (GCU)-trnG* | 788 | 91 | 26 |

**Table S6.** Pairwise comparisons of *Callicarpa oligantha* collected in this study (*C. oligantha*), *C. brevipes*, *C. oligantha*-TYPE (*C. oligantha* isotype), *C. brevipes*-TYPE (*C. brevipes* lectotype) and *C. kwangtungensis* based on three leaf morphological characters in Adonis analysis using pairwise Adonis package.

| **Pairs** | ***R*^2^** | ***P*-value** | ***P*-adjusted** |
| --- | --- | --- | --- |
| *C. oligantha* vs. *C. oligantha-*TYPE | 0.0177 | 0.583 | 0.5830 |
| *C. oligantha* vs. *C. brevipes* | 0.1733 | 0.067 | 0.0744 |
| *C. oligantha* vs. *C. brevipes*-TYPE | 0.3573 | 0.016 | 0.0267 |
| *C. oligantha* vs. *C. kwangtungensis* | 0.6763 | 0.001 | 0.0050 |
| *C. oligantha-*TYPE vs. *C. brevipes* | 0.1331 | 0.039 | 0.0488 |
| *C. oligantha-*TYPE vs. *C. brevipes*-TYPE | 0.2037 | 0.010 | 0.0200 |
| *C. oligantha-*TYPE vs. *C. kwangtungensis* | 0.5239 | 0.001 | 0.0050 |
| *C. brevipes* vs. *C. brevipes*-TYPE | 0.7586 | 0.005 | 0.0150 |
| *C. brevipes* vs. *C. kwangtungensis* | 0.9156 | 0.006 | 0.0150 |
| *C. brevipes*-TYPE vs. *C. kwangtungensis* | 0.8933 | 0.026 | 0.0371 |

**Table S7.** Pairwise comparisons of *Callicarpa oligantha*, *C. brevipes* and *C. kwangtungensis* collected in this study based on three flower morphological characters in Adonis analysis using pairwise Adonis package.

| **Pairs** | ***R*^2^** | ***P*-value** | ***P*-adjusted** |
| --- | --- | --- | --- |
| *C. oligantha* vs. *C. brevipes* | 0.0160 | 0.587 | 0.5870 |
| *C. oligantha* vs. *C. kwangtungensis* | 0.7992 | 0.001 | 0.0015 |
| *C. brevipes* vs. *C. kwangtungensis* | 0.6066 | 0.001 | 0.0015 |

**Table S8.** *T*-test analyses of three leaf morphological characters among *Callicarpa oligantha*, *C. brevipes*, *C. oligantha*-TYPE (*C. oligantha* isotype), *C. brevipes*-TYPE (*C. brevipes* lectotype) and *C. kwangtungensis*.

| **Pairs** | **Leaf length (cm)** | **Leaf width (cm)** | **Leaf petiole length (cm)** |
| --- | --- | --- | --- |
| *C. oligantha* vs. *C. brevipes* | 0.4800 | 0.2400 | 0.5600 |
| *C. oligantha* vs. *C. oligantha-*TYPE | 0.7300 | 0.0002 | 0.7600 |
| *C. oligantha* vs. *C. brevipes*-TYPE | 0.0059 | 0.6600 | 0.4900 |
| *C. oligantha* vs. *C. kwangtungensis* | 0.0015 | 0.0015 | 0.0029 |
| *C. brevipes* vs. *C. oligantha-*TYPE | 0.2200 | 0.0015 | 0.4700 |
| *C. brevipes* vs. *C. brevipes*-TYPE | 0.0043 | 0.9100 | 0.0810 |
| *C. brevipes* vs. *C. kwangtungensis* | 0.0006 | 0.0002 | 0.0085 |
| *C. oligantha-*TYPE vs. *C. brevipes*-TYPE | 0.0095 | 0.0095 | 0.2600 |
| *C. oligantha-*TYPE vs. *C. kwangtungensis* | 0.0095 | 0.0095 | 0.0095 |
| *C. brevipes*-TYPE vs. *C. kwangtungensis* | 0.0029 | 0.0029 | 0.0290 |

**Table S9.** *T*-test analyses of three flower morphological characters among *Callicarpa oligantha*, *C. brevipes* and *C. kwangtungensis*.

| **Pairs** | **Inflorescence width (cm)** | **Inflorescence branching** | **Filament length (cm)** |
| --- | --- | --- | --- |
| *C. oligantha* vs. *C. brevipes* | 0.3600 | 0.8900 | 0.79 |
| *C. oligantha* vs. *C. kwangtungensis* | 0.0014 | 0.0008 | 0.77 |
| *C. brevipes* vs. *C. kwangtungensis* | 9.4e-05 | 8.3e-05 | 0.67 |

**Fig. S1** Species tree generated using StarBEAST2 based on eight cpDNA regions. The number above each branch represents posterior probability.

**Fig. S2** Specimens of *Callicarpa oligantha* and *C. brevipes* and all specimens are deposited in the herbarium of the College of Agriculture, Guangxi University (GAUA). a. *C. oligantha* (No. WeiCX11); b. *C. oligantha* (No. ZYC312); c. *C. oligantha* (No. WeiCX14); d. *C. oligantha* (No. WeiCX13); e. *C. oligantha* (No. WeiCX12); f. *C. brevipes* (No. 6); g. *C. brevipes* (No. 078); h. *C. brevipes* (No. 082); i. *C. brevipes* (No. 0150); j. *C. brevipes* (No. LB0169).

**Fig. S3** Stellate indumentum of *Callicarpa oligantha* and *C. brevipes* on leaves and fruits*.* a-b. Adaxial leaf surface of *C. oligantha*. c-d. Abaxial leaf surface of *C. oligantha*. e. fruits of *C. oligantha*. f-g. Adaxial leaf surface of *C. brevipes*. h-i. Abaxial leaf surface of *C. brevipes*. j. fruits of *C. brevipes*.
